# Information Retrieval during Free Listing Is Biased by Memory: Evidence from Medicinal Plants

**DOI:** 10.1371/journal.pone.0165838

**Published:** 2016-11-04

**Authors:** Daniel Carvalho Pires de Sousa, Gustavo Taboada Soldati, Julio Marcelino Monteiro, Thiago Antonio de Sousa Araújo, Ulysses Paulino Albuquerque

**Affiliations:** 1 Laboratório de Ecologia e Evolução de Sistemas Socioecológicos (LEA), Departamento de Biologia, Universidade Federal Rural de Pernambuco, Av. Dom Manoel de Medeiros, s/n, Dois Irmãos, 52171–900, Recife, Pernambuco, Brasil; 2 Universidade Federal de Juiz de Fora, Rua José Lourenço Kelmer, s/n, Martelos, 36036–330, Juiz de Fora, Minas Gerais, Brasil; 3 Universidade Federal do Piauí, Campus Universitário Ministro Petrônio Portella, s/n, Ininga, 64049–550, Teresina, Piauí, Brasil; 4 Laboratório de Produtos Naturais (Lapronat), Departamento de Farmácia, Universidade Federal de Pernambuco, Av. Professor Moraes Rego, n° 1235, Cidade Universitária, 50670–901, Recife, Pernambuco, Brasil; University of Illinois-Chicago, UNITED STATES

## Abstract

Free listing is a methodological tool that is widely used in various scientific disciplines. A typical assumption of this approach is that individual lists reflect a subset of total knowledge and that the first items listed are the most culturally important. However, little is known about how cognitive processes influence free lists. In this study, we assess how recent memory of use, autonoetic and anoetic memory, and long-term associative memory can affect the composition and order of items in free lists and evaluate whether free lists indicate the most important items. Based on a model of local knowledge about medicinal plants and their therapeutic targets, which was collected via individual semi-structured interviews, we classify each item recorded in free lists according to the last time that the item was used by the informant (recently or long ago), the type of relevant memory (autonoetic or anoetic memory) and the existing associations between therapeutic targets (similar or random). We find that individuals have a tendency to recall information about medicinal plants used during the preceding year and that the recalled plants were also the most important plants during this period. However, we find no trend in the recall of plants from long-term associative memory, although this phenomenon is well established in studies on cognitive psychology. We suggest that such evidence should be considered in studies that use lists of medicinal plants because this temporal cognitive limit on the retrieval of knowledge affects data interpretation.

## Introduction

Among the data collection techniques used in different studies, free listing stands out because it provides objectivity and speed in the characterization of the local knowledge of various cultural domains [1,2]. Cultural domains are shared sets of terms that have the same degree of conceptual contrast (e.g., domestic animals or types of fruit) [3], and their identification through free listing allows the development of studies in distinct scientific disciplines [4–11], including ethnobiology [1,12–17]. The method involves asking each individual using an objective and simple question on the knowledge to be characterized (e.g., "Which 'x' do you know?," where "x" is the cultural domain being evaluated) [2,8,10,18], resulting in several individual lists with the amount of knowledge about a particular domain.

Free lists can be used to support different approaches and also provide insights on local knowledge, particularly when these approaches are based on the assumption that the first and most frequently mentioned items on the list are also the most culturally significant [1,2,10,17], allowing the identification of the most salient items of a particular culture [19,20]. However, little is known about the variables that determine recall processes during free listing and consequently the order and frequency of the mentioned items. Given that ethnobiology uses data from free lists to compare knowledge regarding medicinal plants from different locations [13,15,21–23], characterize items with bioprospecting potential [12,16], evaluate the resilience of knowledge about local medical systems [14,24,25], study patterns of use relative to plants’ organoleptic properties [26], or simply characterize local knowledge about natural resources [27], an understanding of the nature of the information in free lists may allow for the identification of possible biases related to free listing.

Previous studies on cognitive psychology have indicated that the human mind generally works using three main independent processes: designated encoding, storage, and retrieval of information [28–30]. When we experience any one event, some of its references are encoded, triggering a series of cognitive processes to store these events in stable memory traces, thus structuring our long-term memory. [31] The recall of these events and the efficiency of this process primarily depend on the similarities between the references used in the recall (encoding of present stimuli) and those used in storage (encoding of past stimuli) [32,33]. For example, we produce larger lists of plants when we are in a plant shop because of the amount of visual references to this knowledge at that moment of the interview. [34]. We understand that the investigation of the nature of memory can greatly enrich our understanding of the individual effects on the responses that are evoked by free listing.

Another cognitive process that influences the type of recall is designated autonoetic consciousness, which allows memories to be experienced using a special state of consciousness of “reliving the moment" (e.g., recalling and re-experiencing the first days of school) [29,35]. The absence of this autobiographical feeling is known as anoetic consciousness, in which, although it is known that the learned content occurred in a previous spatial-temporal context, the special state of reliving the past is not present in the memory (e.g., knowing how to calculate an equation) [29]. Thus, for our study, we believe that the long-term system is neutral; depending on whether the state of consciousness associated with this content is autonoetic or anoetic [29,36].

One of the most popular tools that is used to investigate the biases of information retrieve is the free recall technique [37]. Similar to free listing [10], free recall primarily consists of inviting individuals to recall a list of items stored at an earlier time [35,38]. Studies based on this technique are often manipulated to address spatial-temporal and associative elements of human memory by examining the effects of time between the studies and the similarity between the items on the lists recalled [39,40]. Studies suggest that people have the tendency to recall recent information [32,37], that the spatial-temporal context (autonoetic consciousness) is more important than anoetic factors for the storage and retrieval of stored lists [29,32,40], and that people use associative semantic information to increase recall efficiency for a sequence of items [37,39–41].

It is reasonable to assume that these cognitive processes of recall operate while we collect information about the cultural domains during free listing [10]. Thus, using the local knowledge of medicinal plants and their therapeutic targets as the model of information recall, our study aims to identify which of these biases (recent memory of use, autonoetic memory, anoetic memory, and associative memory) influence free listing, both in the order (the sequence of recalled items) and in the composition (the recall of all items) of items.

Therefore, considering the order of mention of the items in free lists, we hypothesize the following: (H1) *the order of the items in the free list is associated with the recent memory of use*, such that (P1) *the first items are used more recently than the last items*; (H2) *the order of the items in the free list is associated with autonoetic memories*, such that (P2) *the first items are more closely related to autonoetic memories than the last items*; and (H3) *the order of the items in the free list is associated with associative memory processes*, such that (P3) *the total number of recalled items associated with similar therapeutic targets in a free list is larger than the total number of items remembered at random (different therapeutic targets)*. Considering the composition of the items in the free lists, we hypothesize the following: (H4) *the items present in a free list are associated with recent memories of use*, such that (P4) *the items mentioned in a free list have more recent memories of use than old memories*; and (H5) *the items present in a free list are associated with autonoetic memories*, such that (P5) *the items mentioned in a free list have more autonoetic memories than anoetic memories*. In addition, to assist in data interpretation, we evaluate whether the recalled items can be considered the most locally important. These data allow us to contextualize our findings and methodological implications.

## Materials and Methods

### Characterization of the study site

The study was conducted in the rural community of Carão, which is located in the municipality of Altinho (8°29'32'' S and 36°03'03'' W), state of Pernambuco, northeastern Brazil, 163.1 km from Recife, the state capital [42]. Altinho has an area of 454,484 km^2^ and a population of 22,353 inhabitants, of whom 12,776 live in urban areas and 9,577 live in rural areas. The weather is hot and semiarid, corresponding to type BSh in the Köppen scale. The average annual temperature is 23.1°C; the average rainfall is 622 mm, with the rainy season being concentrated between June and July. The predominant vegetation is Caatinga [43–45]. It has a dry season of seven to nine months annually, with heavy rainfall occurring in the remaining months [46].

The community of Carão (08°35'13.5'' S and 36°05'34.6'' W) is located 16 km from the center of the municipality of Altinho [16]. At present, the community has approximately 155 inhabitants living in 55 households, according to the data provided by the health center in the first week of March 2015. The area of Carão is primarily rural; its economic activity is based on family farming with the cultivation of several crops, particularly corn, bean, and cassava [43]. These crops supply the basic needs of the families and animals, and the surplus is sold in the municipal market of Altinho [44]. The downtown area is partially paved; however, the dirt roads are the main travel routes among residents. All of the houses are made of brick and are supplied with electricity and rainwater catchment systems. There is no sanitation system, and all waste that is produced by the population is disposed of in landfills. The health care for the inhabitants is provided by a local medical clinic, which is open daily in the morning and afternoon and contains basic first aid materials. There is an elementary school (1^st^ to 5^th^ grades) in Carão, but children must attend middle school (6^th^ to 9^th^ grade) in Altinho if they wish to continue their education [43].

We chose this community because our research group has already conducted a large number of ethnobiological studies in this region, with the goal of characterizing the local medical system [13,47–49]. These studies also established a strong and trusting relationship between our group and the members of the community, facilitating access to the region and to verbal information from community residents. We also selected Carão due to its small population size, which allowed for access to almost all of its residents; thus, this community was an excellent model for study and monitoring.

### Ethical and legal aspects

The first step in the study was to contact the representatives of Carão to discuss the goals and aims of the study and the preparation of the informed consent form. We visited the homes of the participants to explain the study objectives and procedures. Those interested in participating and older than 18 years of age were asked to sign the consent form, according to the legislation in force at the time (Resolution 466/12 of the National Health Council). This study was submitted to and approved by the Research Ethics Committee of the Federal University of Pernambuco (Universidade Federal de Pernambuco—UFPE), under Certificate of Submission for Ethical Review (Certificado de Apresentação para Apreciação Ética—CAEE) No. 46774615.6.0000.5207, and by the Biodiversity Authorization and Information System (Sistema de Autorização e Informação em Biodiversidade—SISBIO).

### Procedures

The data were collected using semi-structured interviews with a primary focus on the free listing method [1] and involved all community residents older than 18 years who agreed to participate (see Supporting Information). Before the interviews, we collected basic socioeconomic information (name, age, gender, educational level, and marital status) to identify the informants. We used knowledge on ethnospecies of medicinal plants as a model because we believe that individual knowledge is retrieved during free listing and that the same informant will not provide different names for the same plant species [50]. This approach generates several lists of items that may or may not be valid for a given culture [2]; however, we realize that the information obtained during an interview results from specific memory processes. Furthermore, most studies in ethnobiology have addressed this type of cultural domain in Latin America [51], including Brazil [52].

The initial interview question was, "What medicinal plants do you know?" This list allowed the identification of the medicinal resources that were known to the informants, particularly the order of mention. After the completion of this list, for each plant mentioned, we asked whether the informant “had used this plant or witnessed someone using this plant as medicine". If the answer was "yes," then we considered that the recall of the plant species was associated with the autonoetic consciousness because the event of use was experienced by the informant. Therefore, we classified this recall as autonoetic memories. If the answer was "no," then we considered that the recall occurred without a sense of "reliving the past" and, therefore, this recall presented anoetic characteristics. We classified these recalls as anoetic memories. For each autonoetic memory, we asked the following: "When was the last time you used this plant or saw someone using this plant as medicine?" We categorized the responses of the informants into two types: recent memories, in case the event had occurred within one year, and old memories, in case the event had occurred more than one year ago.

To assess whether the order of the listed items was influenced by the associative retrieval of the recalled items, we recorded the corresponding therapeutic target for each plant, using it as a model of an "associative cue" of meaning. Our choice of using therapeutic targets as cues is justified by a large number of different possible "plant/therapeutic target" associations that the individual may recall, making it more discriminatory. For our study, we considered both the symptoms of the disease and the disease itself as therapeutic targets because the opinions on disease that were expressed by the residents did not always agree with modern biomedical findings [24].

To investigate whether the recalled items could be considered more locally important, we interviewed the participants using the ranking technique (which consists of asking the participants to list certain items in order of importance) six months after the end of data collection [1,2]. A six-month period was determined based on logistical aspects of our study, and participants were asked to rank items according to the list that they had generated during the first interview. To that end, the list of plants that was generated by each participant at the beginning of the study was randomized to prevent the repetition of the positions.

We interviewed 96 inhabitants over 18 years of age who agreed to participate in the interview, and we applied the ranking technique to 86 participants. These participants listed 117 different ethnospecies of medicinal plants; the total number of items cited by all respondents was 892 ranked items, which represented an average of approximately nine plants per free list per participant.

### Data analysis

To test our first and second hypotheses (the order of the items in a free list is associated with recent and autonoetic memories), we first divided the amount of items generated for each informant into two categories, "the first half of the list" and “the second half of the list”. For example, if an informant listed 10 known medicinal plants, the first half of the list would include the first five plants recalled, and the second half of the list would include the last five plants recalled. We compared the first half with the second half to assess whether the studied cognitive factors influenced the recall of the first half of the list of plants because free listing also assumes that the first items listed are the most culturally important [2]. If the number of items generated individually were odd, we removed the information for the plant found in the middle of the list to simplify the analysis (i.e., if the informant listed seven items, we used the first three and the last three plants in the analysis). To test whether the individual item orders were influenced by recent memories of use, we counted the number of items categorized as recent in the first and second halves of the list. To assess whether the individual item orders were influenced by autonoetic memories, we recorded the number of items categorized as autonoetic in the first and second halves of the list. We discarded the lists with a number of mentions smaller than the number present in the first quartile (N = 6) of our sample for the first two hypotheses because the lists with few mentions could skew the results of our analyses. Thus, the data from 71 of the 96 lists were analyzed using the paired Wilcoxon test.

For our third hypothesis (the order of the items in the free list is associated with associative memory processes), we investigated the sequences of individual mentions of each plant/therapeutic target to identify the formation of groups related to similar therapeutic targets at the time of recall (similar associative cues). Therefore, we defined the plants listed in sequence with at least one therapeutic target in common (regardless of the number of targets) as a similar sequence and the plants listed in sequence with different therapeutic targets as a random sequence. For example, if the informant listed plants in the sequence (plant/therapeutic target) “1/A, 2/A, 3/B, and 4/C,” then we counted each sequence of plants with the same therapeutic target (from 1/A to 2/A = one similar sequence) and each sequence of plants with different therapeutic targets (from 2/A to 3/B and from 3/B to 4/C = two random sequences). At the end of this process, we found all of the similar sequences and all of the random sequences for each informant. We used the Wilcoxon test to assess whether the items mentioned in a free list had more memories of similar sequences than random sequences.

For our fourth hypothesis (the items in the free list are associated with recent memories of use), we counted all of the items related to recent memories and all of the items related to old memories in each list. We then used the Wilcoxon test to assess whether the lists were primarily composed of recent memories at the expense of old memories.

For our fifth hypothesis (the items in the free list are associated with autonoetic memories), we counted all of the items related to autonoetic memories and all of the items related to anoetic memories in each list. The Wilcoxon test was then used to assess whether the lists contained more autonoetic memories than anoetic memories.

To evaluate whether the recalled items could be regarded as the most locally important items, we determined the salience of the items in each free list using ANTHROPAC^®^ software, version 1.0. After data collection, we conducted the Spearman correlation test between the pairs of salience values obtained with the free listing and ranking techniques for each medicinal plant.

All data were analyzed using BioEstat 5.0 software (Ayres et al. 2007), and p-values of less than 0.05 were considered significant.

## Results

We found that the order of the items in the free lists is influenced by recent memories of use (Z = 2.0107, p = 0.0222) and autonoetic memories (Z = 2.0193, p = 0.0217), which led to the confirmation of our first two hypotheses. However, the order of the items in free lists was not influenced by the formation of groups of items with similar therapeutic targets (Z = 7.4871, p < 0.0001; the p-value was related to the total number of random sequences), which negates our third hypothesis. The descriptive statistics for this data set are shown in Table 1. Furthermore, the composition of the lists was influenced by two of the cognitive factors studied, i.e., recent memories of use (Z = 4.1247, p < 0.0001) and autonoetic memories (Z = 8.1929, p < 0.0001). The descriptive statistics for this data set are shown in Table 2.

**Table 1:** Cognitive factors and median, mean, and standard deviation of the number of mentions in free lists applied to informants in the community of Carão, state of Pernambuco (northeastern Brazil), between February and October of 2015. H = hypothesis tested.

Cognitive factors	Median	Mean	Standard deviation
(H1) Recent memories–first half of the list	3	2.9014	2.0505
(H1) Recent memories–second half of the list	2	2.4225	2.0679
(H2) Autonoetic memories–first half of the list	4	4.3944	2.0247
(H2) Autonoetic memories–second half of the list	4	4.0704	2.2635
(H3) Similar sequences	2	1.9053	1.76901
(H3) Random sequences	6	6.5158	3.9353

**Table 2:** Cognitive factors and median, mean, and standard deviation of the number of mentions in free lists applied to informants in the community of Carão, state of Pernambuco (northeastern Brazil), between February and October of 2015. H = hypothesis tested.

Cognitive factors	Median	Mean	Standard deviation
(H4) Recent memories (total)	4	4.6563	3.5387
(H5) Autonoetic memories (total)	6	7.2188	4.2307

In addition, there was a strong correlation between the salience of the items listed during free listing and the salience obtained after the items were ranked by importance, according to the criteria of our informants (t = 18.7491, p < 0.0001, number of pairs = 117). The salience values of each data set are shown in Table 3. Therefore, we believe that free listing characterizes the items that are considered to be the most important by the local people.

**Table 3:** List of medicinal plants, their salience values in free lists, and their ranking, as determined in the community of Carão in the state of Pernambuco in northeastern Brazil.

Medicinal plants	Salience	Ranking
**Aroeira**	0.545	0.619
**umburanaçu**	0.284	0.332
**erva-cidreira**	0.273	0.195
**angico**	0.236	0.205
**capim-santo**	0.236	0.207
**caju-roxo**	0.21	0.262
**jucá**	0.164	0.149
**hortelã-pequeno**	0.159	0.138
**bom-nome**	0.149	0.137
**juá**	0.142	0.133
**jatobá**	0.132	0.175
**babosa**	0.126	0.154
**quixaba**	0.115	0.152
**catingueira**	0.104	0.103
**jurema-preta**	0.097	0.08
**hortelã-grande**	0.088	0.071
**romã**	0.083	0.105
**eucalipto**	0.077	0.064
**hortelã**	0.066	0.048
**mororó**	0.066	0.059
**pinhão-brabo**	0.065	0.071
**podaico-roxo**	0.063	0.037
**boldo**	0.059	0.07
**baraúna**	0.053	0.059
**umbu**	0.052	0.035
**pinha**	0.05	0.032
**goiaba**	0.047	0.048
**alecrim**	0.045	0.042
**erva-doce**	0.042	0.029
**arruda**	0.041	0.028
**marmeleiro**	0.041	0.033
**colonia**	0.037	0.025
**pinhão**	0.033	0.046
**quina-quina**	0.033	0.036
**pega-pinto**	0.032	0.035
**quebra-pedra**	0.032	0.024
**piranha**	0.031	0.021
**jurema-branca**	0.03	0.038
**cedro**	0.029	0.007
**mastruz**	0.026	0.029
**mamão**	0.025	0.008
**mulungu**	0.025	0.037
**umburana-de-cheiro**	0.025	0.028
**jurubeba**	0.024	0.013
**mandacaru**	0.024	0.034
**velame**	0.023	0.03
**alho**	0.021	0.026
**camomila**	0.021	0.015
**cana-de-macaco**	0.021	0.018
**espinheiro**	0.02	0.02
**laranja**	0.019	0.005
**rama-branca**	0.019	0.02
**cana**	0.017	0.02
**jabuticaba**	0.017	0.024
**umburana-braba**	0.015	0.013
**goiaba-branca**	0.014	0.025
**moleque-duro**	0.014	0.016
**batata-de-purga**	0.013	0.018
**cabeça-de-nego**	0.013	0.024
**limão**	0.013	0.014
**alecrim-caco**	0.012	0.001
**aveloi**	0.012	0.011
**carrapixo**	0.012	0.015
**pitanga**	0.012	0.009
**quiabo**	0.012	0.005
**alfazema-de-cabloco**	0.011	0.018
**jurema-lisa**	0.011	0.011
**palho-roxo**	0.011	0.001
**pau-d’arco**	0.011	0.006
**algodão-preto**	0.01	0.004
**anador**	0.01	0.012
**barbatimão**	0.01	0.023
**berinjela**	0.01	0.002
**calumbinho**	0.01	0.01
**cumaru**	0.01	0.005
**gogoia**	0.01	0.006
**graviola**	0.01	0.021
**sacatinga**	0.01	0.01
**benzedeira**	0.009	0.003
**maracujá-de-estralo**	0.009	0.013
**acerola**	0.008	0.005
**caibeira**	0.008	0.008
**louro**	0.008	0.009
**facheiro**	0.007	0.006
**pinhão-roxo**	0.007	0.014
**pirim**	0.007	0.001
**sabugueira**	0.007	0.007
**urtiga**	0.007	0.002
**cardo-santo**	0.006	0.001
**manjericão**	0.006	0.003
**munçambe**	0.006	0.008
**sasafrai**	0.006	0.004
**sena**	0.006	0.006
**alfavaca**	0.005	0.004
**coco-catole**	0.005	0.007
**incó**	0.005	0.004
**couve**	0.004	0.004
**erva-verde**	0.004	0.006
**melão**	0.004	0.002
**melão-miudo**	0.004	0.004
**pau-d’alho**	0.004	0.004
**chuchu**	0.003	0.009
**mentraste**	0.003	0.005
**pimenta**	0.003	0.001
**pinhão-manso**	0.003	0.001
**algaroba**	0.002	0.002
**ameixa**	0.002	0.006
**cabraíba**	0.002	0.013
**cebola-branca**	0.002	0.031
**gengibre**	0.002	0.007
**mentui**	0.002	0.007
**vassoura-de-botão**	0.002	0.001
**apararaio**	0.001	0.012
**coco**	0.001	0.005
**melancia**	0.001	0.001
**mirasol**	0.001	0.003
**papaconha**	0.001	0.003

## Discussion

Our results indicate that recent memories of use and autonoetic memories influence the formation of individual free lists of medicinal plants, either in the mention of the plants initially recalled by the informants (order) or in the total number of items in the lists (composition).

Free recalls can be organized to understand the spatial and temporal variables of human memory. With regard to the confirmation of our two initial hypotheses (recent memories and autonoetic memories), it has been demonstrated that humans have a tendency to initially recall the most recently stored items [37] and that the recall accuracy is significantly higher when information is associated with spatial-temporal autonoetic consciousness, which can influence up to 80% of the responses obtained in the free recall tests [40]. The free lists revealed informants’ tendencies to recall items via recent memories of use and autonoetic memories.

There is evidence of the involvement of other cognitive processes in memory that could influence the responses of informants during free listing. In this context, the testing effect is the phenomenon in which the information more frequently accessed by individuals is also the information that has a greater tendency to be retrieved, being constantly reactivated and fixed in memory [28,53]. Moreover, some studies argue that the long-term system can maintain a set of active recall contents, designated working memory (formerly short-term memory), which operate as efficient cues for the information consolidated in memory [32,33,54,55]. This system of “active” memory maintains the continuity of complex brain activities, allowing efficient access to relevant information during the performance of daily activities (e.g., remembering to prepare a medicine when one needs to prepare it or knowing which treatment to use for a particular disease) [54]. Therefore, because of this tendency to remember constantly retrieved information and because we maintain a set of active information to increase the efficiency of the tasks we perform on a daily basis, we believe that the plant species that are part of the routine treatment of diseases in a community are those with a higher tendency to be recalled during free listing.

We suggest that studies that characterize the knowledge on plants using free listing take into account this aspect of cognition discussed above. In this respect, ethnobiological data that are collected using this tool are specific to a given context, and because local knowledge is dynamic, the interpretations arising from these particular collections must be relativized. Therefore, the theoretical, methodological, and practical interpretations of studies that use free listing should consider that the temporal limit of this tool regarding the local knowledge of medicinal plants is approximately one year and that the representation of the entire body of information developed and selected locally is not feasible.

With regard to the third hypothesis related to associative responses, we found no tendency of the informants in grouping the mentions of medicinal plants during free listing according to the semantic memory of their therapeutic targets. Studies on cognitive psychology that use free recall have indicated that the memories of the informants are also influenced by the associative power of information at the time of recall [37,38,56]. However, each individual makes associations for specific information stored in cognitive structures because the consolidation of this information is affected by several cognitive factors at the time of storage and by individual daily habits and behaviors that can operate during the recall of the items in memory [57]. For example, it is possible that there is no consensus on the recall cue adopted in our study, the therapeutic target, as a recall guide for the formation of groups of plants mentioned by most respondents because medicinal plants may have many intrinsic aspects that can be perfectly used by the individual for the formation of these groups, including organoleptic properties, type of use, and collection site. In addition, the memories can be influenced by the context at the time of the interview, with different environments generating various types of recalls in free lists of plants [34]. Therefore, we suggest that future studies investigate the influence of cognition in the sequence of the items generated by the informants by using other types of associative factors for understanding the nature of the item recall process during free listing.

Furthermore, our correlation analysis indicates that the lists can retrieve the items considered more important by the local population at a certain moment. This result indicates that, despite the influence of cognition on the responses of the study participants, free listing can recall medicinal plants that are known by a group of individuals according to the importance of these items to each individual. This result corroborates the assumption defended by Quinlan [17] regarding the importance of the items listed in the first positions in a free list, and this importance has been considered by several studies that have used this tool to characterize the more salient items in a particular culture; however, these studies not considered a temporal variation in the communities evaluated. In this sense, the local importance of plants, accessed by free listing, seems to be determined by the recent and continuous use and life experiences.

We considered that the answers that were provided during free listing can have autonoetic and anoetic characteristics, which involve responses recalled with the spatial-temporal information of use (by them or by a third party) and those recalled without an associated use. However, individuals can recall the same elements using autonoetic consciousness or not [29,35]. For example, the informant can recall a plant species using autonoetic memory without necessarily having experienced its use (i.e., one may have remembered having read somewhere that such plants can be considered medicinal and may have retrieved this memory at the time of free listing). Considering that all of the responses identified as autonoetic (related to the recall of use) also had a temporal reference of the last use and because we assume that individual free listing is primarily produced by the autonoetic memory [32,40], we believe that this limitation did not influence the responses to our questions.

## Conclusion

Our results indicated that the order of free lists is affected by spatial-temporal references, which have previously been evaluated in studies on cognitive psychology. In other words, the participants can recall items that were experienced, i.e., that have a spatial-temporal context of use involved in the recall. Moreover, our individual analysis of temporal references in this context indicated that the recalled items are items that were experienced during the prior year. Therefore, future studies that use free lists should consider these results because we found that these individual influences affect the responses during free listing and allow the characterization of the medicinal plants used locally only within one year.

In addition, we not found a tendency in the grouping of mentions of medicinal plants according to semantic memory cues of their therapeutic targets, despite the entire body of evidence from studies in cognitive psychology on the influence of associative factors on recall. We understand that the refutation of our third hypothesis may be due to the recall cue that we used in this study or perhaps the lack thereof. Several factors can influence individuals’ priming (a encoding phenomenon that increases processing efficiency of information storage and retrieval [33,38,58–60]) during the recall process; therefore, future studies should address specific recall cues and a larger number of associations to elucidate the influence of cognition on the responses of informants.

Cognitive memory processes are independent from free listing. A methodological limitation of our study, which is a direct consequence of the memory of the informants rather than the application of the tool itself, relates to whether the informants knew any other type of information similar to the cited information [2,8]. In addition, we noted that due to cognitive influences, items recalled during free listing are the most important for the examined population, although only during the preceding year. To the best of our knowledge, our study is the first to investigate whether memory biases can affect the responses during free listing in regard to the spatial and temporal context and also the associative factors that are related to the recall. Several cognitive factors of memory that have been studied in cognitive psychology can be used to understand how cognition affects the recall of the ethnobiological information that depends on the individual responses of the informants.

## Supporting Information

S1 Data Raw data used to perform all analysis. (XLSX)
